# Migratory birds can extract positional information from magnetic inclination and magnetic declination alone

**DOI:** 10.1098/rspb.2024.1363

**Published:** 2024-11-13

**Authors:** Florian Packmor, Dmitry Kishkinev, Thomas Zechmeister, Henrik Mouritsen, Richard A. Holland

**Affiliations:** ^1^ School of Environmental and Natural Sciences, Bangor University, Bangor, Gwynedd LL57 2UW, UK; ^2^ Lower Saxon Wadden Sea National Park Authority, Wilhelmshaven 26382, Germany; ^3^ School of Life Sciences, Keele University, Newcastle-under-Lyme, Staffordshire ST5 5BG, UK; ^4^ Biological Station Lake Neusiedl, Illmitz 7142, Austria; ^5^ Research group ‘Neurosensorik/Animal Navigation’, Institute of Biology and Environmental Sciences, University of Oldenburg, Oldenburg 26129, Germany; ^6^ Research Center for Neurosensory Sciences, University of Oldenburg, Oldenburg 26129, Germany

**Keywords:** bird navigation, magnetoreception, magnetic map, magnetic compass, map-and-compass concept, Eurasian reed warbler

## Abstract

Migratory birds are able to navigate over great distances with remarkable accuracy. The mechanism they use to achieve this feat is thought to involve two distinct steps: locating their position (the ‘map’) and heading towards the direction determined (the ‘compass’). For decades, this map-and-compass concept has shaped our perception of navigation in animals, although the nature of the map remains debated. However, some recent studies suggest the involvement of the Earth’s magnetic field in the map step. Here, we tested whether migratory songbirds, Eurasian reed warblers (*Acrocephalus scirpaceus*), can determine their position based on two magnetic field components that are also associated with direction finding, i.e. magnetic inclination and magnetic declination. During a virtual magnetic displacement experiment, the birds were exposed to altered magnetic inclination and magnetic declination values that would indicate a displacement from their natural migratory corridor, but the total intensity of the field remained unchanged, creating a spatial mismatch between these components. The response was a change in the birds’ migratory direction consistent with a compensatory re-orientation. This suggests that birds can extract positional as well as directional information from these cues, even when they are in conflict with another component of the magnetic field. It remains to be seen whether birds use the total intensity of Earth’s magnetic field for navigation.

## 1. Introduction

Many animals that range or migrate over large distances show a remarkable ability to correct for large-scale displacements from places they have never been to and return to their desired destination, often over hundreds or even thousands of kilometres [1–4]. Such remarkable navigational feats are presumed to be based upon cues detected at the displacement site alone, without reference to any outward journey information or cues emanating from the respective destination and have been called ‘true navigation’ [5–7]. Following Kramer [8,9], it is conventionally thought that the navigation mechanism that animals use approximates to a map and a compass applied in a two-step process (map-and-compass concept) [8,10], whereby they first detect their position (the map step) and then take up the compass direction towards their goal (the compass step). The stars [11], the sun [12] and the Earth’s magnetic field [13] have all been shown to be used as cues for the compass step. The map step is proposed to be based on environmental cues that provide some reliable spatial gradients, which the animals learn during their first migration [14,15]. Using at least two such gradients which would intersect at a reasonably large angle and vary in a large-scale geospatial context would allow animals to fix their position by means of a ‘bi-coordinate map’ [5,14,16]. However, there is no clear agreement on which cues are involved and their specific usage is likely to depend on which phase of a navigational task an animal is currently in (long-distance navigation phase, homing phase or pin-pointing-the-goal phase [15]). Furthermore, there is an ongoing debate over the very nature of navigational maps in animals [14,15,17].

One line of evidence, however, indicates that animals can use components of the Earth’s magnetic field to assess their position in a number of different contexts. Magnetic inclination (the dip angle of the field lines), total magnetic intensity (the combined intensity of the vertical and horizontal components of the magnetic field) and magnetic declination (the difference between the direction to the geographic and magnetic poles) all show geospatial variation that can be used to locate position. Although these components are human constructs for measuring and describing the nature of the Earth’s magnetic field, it does seem that animals respond to artificial changes in these components in a way that is consistent with their use to locate position. For example, studies on hatchling sea turtles, salmon and eels indicate that they can respond to regional magnetic field signatures that they have never experienced in a way that is ecologically relevant, suggesting that a series of inherited magnetic signposts guide their behaviour [18–20]. Alongside this, evidence from homing experiments on newts suggests that magnetic inclination can form at least one coordinate of a magnetic map [21]. However, it remains to be established how temporal variation in the magnetic field, which is greater than the spatial variation at the distances used (1–2 km) can be accounted for in these experiments ([5], see [22] for a possible mechanism). Analyses of ringing data from both songbirds (Eurasian reed warblers, *Acrocephalus scirpaceus*) and procellariiform seabirds (Manx shearwaters, *Puffinus puffinus*) suggest that the magnetic inclination of the field is used as a positional cue indicating the arrival at the respective natal/breeding area by means of an imprinting mechanism [23,24]. Other experiments have demonstrated that birds can use some combination of total intensity and inclination to determine their position on the migratory route and use this as a cue to adjust their behaviour (e.g. adjust the migratory fuelling) [25,26] (but see [27]).

However, a key focus of the use of the magnetic field as a map cue has been to discover whether it can explain the ability of animals to apparently perform true navigation. Virtual magnetic displacement experiments, in which captive animals are subjected to magnetic field conditions simulating a different geographic location, have been employed to study this phenomenon in crustaceans (spiny lobsters), amphibians (newts) and reptiles (turtles), as well as birds [21,28–30]. Experiments have indicated that these animals can use some combination of total magnetic intensity, magnetic inclination and most recently, magnetic declination [31] to locate their position when displaced from the normal migratory path. However, the nature of these virtual displacements left it ambiguous as to whether: (i) in the case of newts and lobsters, the magnetic field alone could provide all cues necessary to locate their position or (ii) in the case of birds, animals were only capable of combining previously experienced values of the magnetic cues in novel combinations to calculate their position [32], or were capable of extrapolating from known values of the magnetic field to completely unknown values never experienced before, as would fit the most comprehensive definition of true navigation [5].

Recent virtual displacement studies with the Eurasian reed warbler, a long-distance migratory songbird, have addressed these questions. Their results suggest that when inclination, declination and total intensity of the magnetic field are changed such that all three correspond to the natural magnetic field at a real geographic location, birds respond as if displaced to that location [30,31]. Even when all three potential cues are combined to correspond to a real location that is outside the known breeding range, and therefore presenting values of the Earth’s magnetic field the birds have never experienced before, they were still able to correct for this virtual magnetic displacement and head towards their natural migratory route [32]. However, when one component (magnetic declination) is changed such that it does not correspond to the others in their known geospatial context, the birds do not respond [32], instead continuing to take their seasonally appropriate migratory direction. This supports a magnetic map that can be extrapolated beyond prior experience. However, it is not clear, at this stage, whether the birds in [32] did not respond to the change in magnetic declination alone because they discarded the magnetic field as unreliable information due to the discrepancy between the components, and simply used their innate heading (as in e.g. [33–35]), whether they simply do not use declination as part of their map or whether they weighed the combination of the two unchanged components corresponding to their current physical location as more reliable than the changed component.

Generally, it is conceivable that birds may weigh the reliability of cues in relation to others and when one does not match the known geospatial context, it may be discarded. While the weighting of three cues may appear simple, the rather complex large-scale geospatial variation of inclination, declination and total intensity of the magnetic field across the Earth’s surface could in practice make it a challenging task. This suggests a more complex cue integration mechanism than a simple ‘bi-coordinate’ gradient map, as has been originally proposed [16]. In order to recognize such a mismatch, the birds would have to have learned the geospatial variation in all the components relative to each other, extrapolate beyond their prior experience and recognize that one (in this case magnetic declination [32]) was out of sync. It also suggests that if it is part of the map, magnetic declination alone does not simply provide a rule of thumb mechanism that helps determine east-west drift independently of other cues but that the complex geospatial variation of the three components in relation to each other may be encoded into the birds true navigation map.

In the current study, our aim was to test whether two of the components derived from the Earth’s magnetic field (i.e. magnetic inclination and magnetic declination) but not the other (i.e. total intensity), can be used alone to indicate position to a migratory bird, or whether effective positioning requires all three cues to match their actual geospatial context on Earth. We, therefore, carried out a virtual magnetic displacement experiment with long-distance migratory songbirds during autumn migration in which magnetic inclination and magnetic declination, but not the total intensity of the magnetic field, were changed to match a different location, in order to determine whether these, at least in some cases, may be sufficient to provide information for the magnetic map.

## 2. Material and methods

### (a) Study species and site

**Figure 1.**
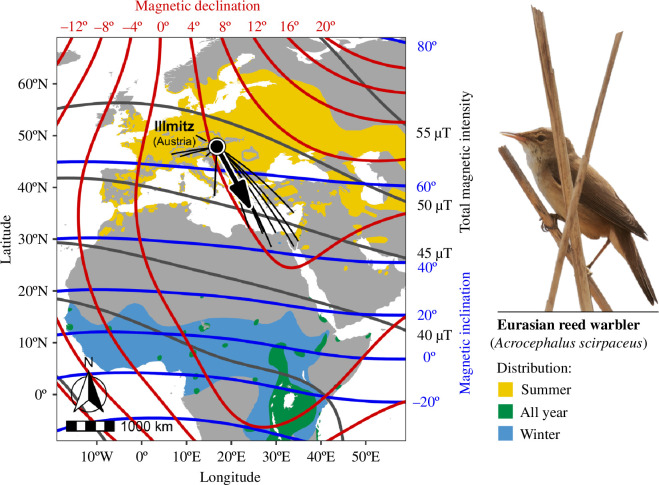
Distribution range of reed warblers across Europe, Northern and Central Africa and the geospatial context of cues derived from the Earth’s magnetic field in these areas. The black dot indicates the location of the study site. The natural migratory direction given in the map (black arrow) has been derived from reed warblers banded at or near the study site. Mean direction: *α* = 156°, 95% confidence interval (CI) = 144−175°, *n* = 19; Rayleigh test of uniformity: *r* = 0.80, *p* < 0.001. The recoveries are depicted as great circle lines (black). The Eurasian reed warblers’ distribution range is shown in yellow (summer), green (all year) and light blue (winter), respectively. Isolines of cues derived from the Earth’s magnetic field, i.e. magnetic inclination (blue), magnetic declination (red) and total magnetic intensity (dark grey) are depicted as solid lines. Eurasian reed warbler distribution data were provided by BirdLife International and the Handbook of the Birds of the World (http://datazone.birdlife.org/species/requestdis). Photo by Flora Bittermann.

Our species of choice for this study was the Eurasian reed warbler, *A. scirpaceus* (reed warbler hereafter), which represent a well-established model species for studies on navigation in night migrating passerine birds [3,30–32,34,35]. Our experiments were conducted at the Biological Station Lake Neusiedl in Illmitz, Austria (47° 46’ 08.9”N, 16° 45’ 57.2”E). Ringing recoveries have established that birds at this site are on the eastern side of the migratory divide and migrate southeast during the autumn (figure 1). We captured 11 adult birds in 2019 and 10 adult birds in 2020, which were held in captivity. The capture, determination of age, bird housing and husbandry were identical to our previous study and full details can be found in that article [32]. All birds were released at the capture site after the completion of the study in the same season.

### (b) Magnetic displacement experiment

All 21 reed warblers included in our virtual magnetic displacement experiment were repeatedly tested in modified Emlen funnels while being subjected to two different magnetic field conditions during their autumn migration season in 2019 and 2020, respectively. First, all birds underwent control orientation tests (20–22 September 2019, and 12–14 September 2020; three tests per individual bird). These tests were performed in the natural magnetic field (NMF) at the test site (NMF: total magnetic intensity~48 730 nT, magnetic inclination~64.3°, magnetic declination~4.6°).

Following this we conducted tests with all birds in a changed magnetic field (CMF) to simulate a displacement (29 September to 1 October 2019, and 15–21 September 2020; three tests per individual bird). Note that the periods used for tests in the NMF in 2019 and CMF in 2020 partly overlap (electronic supplementary material, figure S1). The CMF consisted of total magnetic intensity kept at the natural value of the study site, but the inclination increased by 8.6° and the declination increased by 9.8° to match values naturally found *ca* 2700 km to the ENE at our previously used virtual displacement site, Neftekamsk, Russia [32] (electronic supplementary material, figure S2). Unlike the previous study, where all three cues were changed to match Neftekamsk, the CMF presented to the birds in the current experiment, in fact does not correspond to any NMF found on Earth (electronic supplementary material, figure S3). The NMF found around Neftekamsk features a much higher total magnetic intensity of ~55.510 nT. It would thus test whether birds required all three magnetic parameters to match known spatial context in order to recognize the change as a displacement, or only the magnetic inclination and magnetic declination.

### (c) Magnetic set-up and magnetic field measurements

For manipulating the magnetic field birds experienced during the experiments we used a three-axis, single-wound 2 m × 2 m × 2 m Helmholtz coil. The coil was identical to that used in our previous virtual displacement experiment [32] and full details of the specifications, procedures to measure the magnetic field produced and heterogeneity measurements can be found in that article. We had planned to use a double-wrapped Merritt design coil as recommended in [36], but it was not available for technical reasons. However, the pattern of results from our previous experiment [32] did not suggest that temperature or vibration explained the results, because two different magnetic field manipulations produced different effects in that experiment. Nor did it suggest that radio frequency (RF) field effects [37,38] from the power supplies were an issue because controls were also tested with the power supplies on and oriented in the seasonally appropriate direction.

All tests were conducted in the central area of <1% heterogeneity of the applied field strength (<200 nT). The coils were powered by three DC power supplies (model BOP 50-2M, Kepco Inc., Flushing, NY, USA). To obtain NMF parameter values (including its X-, Y- and Z-components) as given in this study, we queried the NOAA World Magnetic Model (WMM, 2019 and 2020, respectively [39]) using coordinates and altitude (113 m) of our study site near Illmitz and the mean dates of the periods used for our experiments. The CMF parameter values (X-, Y- and Z-components) used for the magnetic displacement were calculated using the inclination and declination of the virtual magnetic displacement site and the total magnetic intensity of our study site (obtained from the NOAA website calculator using the WMM model for 2019 and 2020, respectively [39]) as follows:

X-component = (total magnetic intensity*_s_* * cosine(magnetic inclination*_m_*)) * sine(magnetic declination*_m_*)

Y-component = (total magnetic intensity*_s_* * cosine(magnetic inclination*_m_*)) * cosine(magnetic declination*_m_*)

Z-component = total magnetic intensity*_s_* * sine(magnetic inclination*_m_*)

*s* = study site; *m* = magnetic displacement site

While in the CMF up to 10 birds were kept in a cubic aviary (80 cm^3^) in the area of <1% heterogeneity. When being tested, four Emlen funnels were placed in the same area, above the aviary so that birds never left the area of <1% heterogeneity when they were being transferred from the aviary to the Emlen funnel.

### (d) Orientation tests

Orientation tests were conducted using Emlen funnels and followed the procedure established in [32,40] and were identical to those studies, the latter of which was conducted concurrently with the current study. Full details of the procedure including timing and duration of tests, randomization procedures to avoid temporal and spatial bias in the birds’ position and specifications of the Emlen funnels can be found in those articles. During NMF tests, the power supplies, which were always located in the same place, were turned on, but were not connected to the coil, to control for the exposure to their noise and potential RF fields during testing.

Because the scratch marks that are used to determine the desired direction of birds in Emlen funnel tests are inherently noisy data with an element of subjectivity, it was essential to ensure that observer bias was controlled for. To achieve this, we took advantage of the fact that the inner scratch-sensitive lining of each Emlen funnel has a seam. During tests, the alignment of the funnel seam was alternated in the cardinal compass directions so that these seams were not consistent between birds. The orientation of the birds was then assessed independently by two researchers who were naive to the actual alignment of the funnel and calculated it on the assumption that the funnel seam was aligned to the north. The actual funnel seam direction was then corrected for later. The mean direction of the bird in a given test was calculated as the resultant vector between the two independent assessors, unless the two directions deviated by more than 30° or if the bird left less than 35 scratch marks (a commonly used threshold for Emlen funnel experiments [41]), and only if there was a clear unimodal orientation. Birds were tested three times under each of the two conditions (NMF and CMF). Because individual birds may show migratory activity but the mean direction of the group is not oriented in a seasonally appropriate direction in a ‘pre-migratory period’ [42,43], it was necessary to test birds sequentially with control tests to first establish that the group is oriented in a seasonally appropriate direction. A second-order mean was then calculated from the individual mean directions of each bird for each condition. Only birds that showed a directional preference during at least two of the orientation tests were included in the dataset. Of the 21 birds tested, 14 showed orientations in at least two tests in the NMF and 16 in the CMF and all these results are available in electronic supplementary material, table S3 as well as on Figshare [44]. Of those, 12 birds showed orientation in at least two tests in both conditions (see electronic supplementary material, table S3). Because of the paired design, only birds that showed orientation in both conditions are presented in the orientation analysis.

### (e) Statistical analyses

We used R version 4.0.4 [45] and Oriana version 4 (Kovach computing services, Pentraeth, UK) to conduct analyses of the data.

We used the Rayleigh test for uniformity to establish whether the NMF and CMF distributions were significantly different from a uniform distribution [46]. Additionally, we assessed the likelihood of the 10 models for orientation behaviour described by [47] for the orientation data obtained under the two conditions using the model selection procedure implemented in the package ‘CircMLE’ [48]. We compared the models by means of the corrected Akaike information criterion (AIC_c_; [49]) and the corresponding AIC_c_ model weights. We used the non-parametric Moore’s paired test in Oriana on the birds that showed at least two oriented tests in both conditions to avoid violating the assumption of independent samples in the more commonly used Mardia–Watson–Wheeler test.

Finally, the method for testing orientation in Emlen funnels necessitates a before and after design, due to the fact that it must first be established that the group is expressing seasonally appropriate orientation before experimental manipulations can be performed [42]. This design, however, may bear the risk of an effect of the sequential testing on the results of the experiment, which then could alternatively be explained by a seasonal change in the birds’ orientation and thus, their inherited migratory direction. Such a seasonal change in the birds’ orientation, also known by the term ‘Zugknick’, has been reported for inexperienced individuals of two other migratory songbird species [50–52]. Despite this concern, our previous study in 2018 did not support an effect of time within the season on the observed change in the east-west component of the birds’ orientation established by our model-based analyses [32]. Furthermore, in a study on inexperienced reed warblers tested at the same site, during the same time and later within the season than the experimental birds in this study, we found a consistent orientation towards the southeast for the entire time period relevant to the present study [40], consistent with the expected natural migratory direction (see above) and no support for the presence of a ‘Zugknick’ in this population, at least during the time of our experiments. Nevertheless, the current study provides two further migratory seasons from two different years to add to the model-based analysis in [32]. We, therefore, used the same modelling approach as used in [32] but combined the data from the three seasons in which adult reed warblers were tested (2018, 2019, 2020) to test whether any change in the birds orientation could be explained by the time of testing rather than the experimental treatment. The data used are available in electronic supplementary material, table S4 as well as on Figshare [44] and included all birds that showed oriented behaviour in either condition, not just those that oriented in both conditions. This follows the same approach as reported in [32] and the R code to reproduce the analysis is deposited on Figshare [53]. We could not find a circular package that allowed us to do a mixed model analysis on the raw data, and so, as the orientation of birds was found to change mostly in the east-west direction (from 169° (SSE) to 266° (WSW)), we modelled the effect of time within the season on the sine of the directions measured. The sine of a direction is limited between −1 (sine of 270° (W)) and 1 (sine of 90° (E)). The sine was linearly transformed from its original scale to the open unit interval (0, 1) following [54] by first taking y' = (y – a)/(b – a), where ‘b’ is the highest possible value (1) and ‘a’ is the smallest possible value (−1), and then compressed the range to avoid highest and lowest possible values by taking y'' = (y'(n − 1)+1/2))/n, where ‘n’ is the sample size. This transformation allowed the application of a generalized additive mixed model (GAMM) of the family ‘betar’. The function ‘gam’ implemented in the R package ‘mgcv’ [55] was used to fit the GAMM with the day of year as a smoothing term and the magnetic condition as an additional explanatory factor with two levels: NMF and CMF. The GAMM included the birds’ ID as a random effect to account for non-independence of data from repeated tests of individuals. Furthermore, the GAMM included the year as a further random effect. No serious violations of the models’ assumptions were discovered when checked using diagnostic plots generated with the function ‘gam.check’ implemented in the R package ‘mgcv’ [55].

## 3. Results

**Figure 2.**
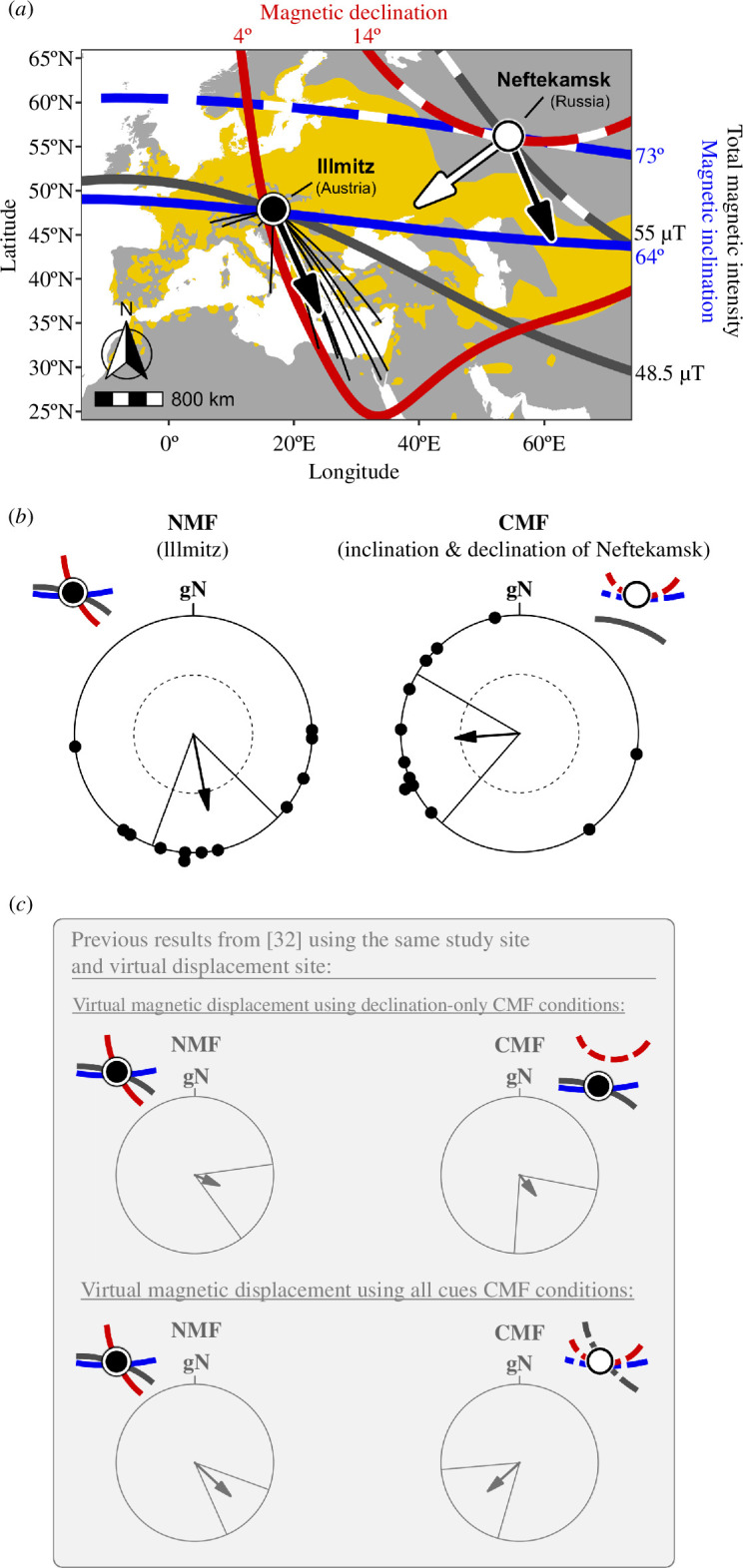
Predicted responses and results for the CMF treatment. (*a*) Map illustrating Eurasian reed warblers’ natural migratory direction (black arrow) from the study site (Illmitz, Austria; black dot) under natural magnetic field (NMF) conditions during autumn and the predicted migratory directions from the virtual displacement site (Neftekamsk, Russia; white dot) under changed magnetic field (CMF) conditions (magnetic inclination and magnetic declination changed), if birds do (white arrow) or do not (black arrow) perceive these magnetic values as a translocation and respond by re-orienting towards the site of capture or the migratory corridor of the population (see figure 1 for map colour key). Dashed isolines give values naturally occurring near the virtual displacement site (see Methods for exact values). (*b*) Orientation of birds tested under NMF conditions of the study site (left) and under CMF conditions (right). Black dots indicate the mean direction of each individual bird tested; arrows show the second-order mean directions and their vector lengths; the inner and outer dashed circles indicate the threshold needed for significance by the Rayleigh test (*p* < 0.05 and *p* < 0.01, respectively); solid lines either side of mean vectors show the 95% confidence intervals. (*c*) Results from our previous virtual magnetic displacements [32]. Both the study site and the virtual displacement site are the same as in the current study.

In the NMF of the study site, Illmitz, the 12 birds that showed orientation in both groups were oriented towards south-southeast (figure 2*a*,*b*; mean direction *α* = 169°, 95% CI 138−200°, *n* = 12; Rayleigh test of uniformity: *r* = 0.651, *p* = 0.004; best described by a unimodal orientation model, see electronic supplementary material, table S1). Subsequently, the same birds were exposed to a CMF with total magnetic intensity kept at the natural value of the study site, but the magnetic inclination increased by 8.6° and the magnetic declination increased by 9.8° to match values naturally found *ca* 2700 km to the ENE at our virtual displacement site, Neftekamsk, Russia (CMF: total magnetic intensity ~48 730 nT, magnetic inclination ~72.9°, magnetic declination ~14.4°; electronic supplementary material, figure S2). In the CMF, these birds shifted their orientation compared with the control direction and were oriented towards the WSW (figure 2*b*; mean direction *α* = 266°, 95% CI 225−307°, *n* = 12; Rayleigh test of uniformity: *r* = 0.544, *p* = 0.025; best described by a unimodal orientation model, see electronic supplementary material, table S1). The two circular distributions obtained under NMF and CMF conditions were significantly different (figure 2*b*, Moores test: R’ = 1.33, *p* < 0.005). This difference is consistent with re-orientation to the site of capture (i.e. study site; direction from the virtual displacement site: 266°) or the natural migratory corridor and corresponds to the response shown when all cues are changed in [32] (figure 2*c*).

The GAMM fitted to test the effect of time within season on individual directions from this data and the data collected for [32] during autumn 2018 did not support an effect of the time within the season on the east-west component of the birds’ orientation (electronic supplementary material, table S2, figure S4 χ2 = 1.08, *p* = 0.3), but only an effect of the (experimentally changed) magnetic conditions (electronic supplementary material, table S2, figure S4: χ2 = 5.52, *p* < 0.001). We, therefore, argue that our current results were not affected by any underlying seasonal pattern in the birds’ directional disposition.

## 4. Discussion

Our current results together with the results of our preceding study [32] suggest that two components derived from the Earth’s magnetic field (i.e. magnetic inclination and magnetic declination) are sufficient for reed warblers to determine their position during autumn migration (figure 2). When we changed magnetic inclination and magnetic declination in a way that still followed their large-scale geospatial context during our virtual magnetic displacement experiment, the birds responded as if they had been displaced to the virtual displacement site and re-oriented towards the initial capture site or their natural migratory corridor.

If magnetic declination was not detected or used by the birds as a positional cue, then the birds would have faced a mismatch between the total intensity of the capture site and a higher magnetic inclination value. As magnetic inclination increases in a northward direction relative to the capture site, the birds’ alternative would have been either to orient in the same direction as at the capture site as indicated by the total magnetic intensity or in a very similar direction (*ca* due south) as expected if they thought they were at a location further north. Orienting westwards would not make ecological sense in either of these situations and so it seems unlikely that the response of the birds in the current study is a non-specific reaction to a magnetic field that is geospatially conflicted. In previous experiments where the magnetic field components did not match [32] or were removed [34], the birds reverted back to orienting in the same direction as at the capture site (i.e. falling back on an innate compass direction [56,57]). We have never seen reverse orientation, which for this study site, would be northwest. Nevertheless, follow-up experiments in which inclination alone was changed would be the next step to establish this. Thus, neither total magnetic intensity, which was not changed to match the virtual displacement site, nor any other putative geospatial cues that were present at the capture and testing sites, appear to have played any part in the navigational process under the given circumstances. In the context of our previous experiments on reed warblers suggesting that magnetic declination can form a part of the navigational map [31] and that this is only true when it matches the geospatial context of the other components of the magnetic field [32], this could be interpreted as follows: (i) any two of the three components derived from the Earth’s magnetic field are sufficient to recognize a change in location. In our experiment, the birds may have ignored or significantly downweighed any positional information provided by total magnetic intensity because it was inconsistent with the other two components that matched their known geospatial context, i.e. essentially a ‘majority rule’ or (ii) the total magnetic intensity of the field does not form part of the magnetic map in reed warblers. Further experiments will be needed to discover which of these is the case. Furthermore, it will also be interesting to see whether the accuracy of the map can be ‘titrated’ as has been performed with newts [21]. At present, we cannot distinguish between a map based on estimating displacement direction alone, in which the birds use a ‘rule of thumb’ based on the direction of the increase/decrease in the field components, or a map encoding both distance and direction, which would be more accurate (see [32] for further discussion of this issue).

Generally, there is still intense debate around the very nature of any navigational map in birds as well as in other animal taxa [5,7,14,15,17,58]. The results of some previous studies have suggested a significant role of the Earth’s magnetic field as a source of positional information during navigation, while others have not. In Gray Catbirds (*Dumetella carolinensis*), e.g. anosmia but not a magnetic manipulation was found to disrupt their ability to correct for a displacement [33], and olfactory but not magnetic cues seem to be crucial for navigation in migrating [59] or homing [17,60–62] seabirds, like gulls and procellariiforms, but see [63,64] for an alternative interpretation. Moreover, a recent virtual magnetic displacement study did not observe any behavioural response to a change in magnetic declination in European robins (*Erithacus rubecula*) and garden warblers (*Sylvia borin*) [65] even though an experiment at the same site observed a clear behavioural response to the same experimental treatment in reed warblers [31].

Nevertheless, a significant body of evidence now supports the use of cues derived from the Earth’s magnetic field for determining position in several bird species [23–27,30–32,42,66–69], and our current results further corroborate the role of a magnetic map for navigation in night-migratory songbirds, using the reed warbler as a main model species. The present study suggests that reed warblers can use a combination of magnetic inclination and magnetic declination alone to determine their approximate position relative to their goal (i.e. map information), but it does not exclude that total intensity can play an important role in other situations [70] or that it is used as part of the birds’ magnetic map when its value is consistent with those of magnetic inclination and/or magnetic declination. It is also possible that total intensity cannot be extrapolated beyond prior experience in the way that inclination and declination are, but that values must have been experienced before being used as part of the map. It is difficult to imagine, however, why two of the cues could be extrapolated while the third is not.

There are studies suggesting that sea turtles can detect total magnetic intensity and use it in combination with magnetic inclination, but not magnetic declination, for determining position during migration [71]. Curiously, however, there is little direct evidence suggesting that birds specifically detect total magnetic intensity (but see [72]), let alone use it for navigation. Most studies that reported behavioural responses did not isolate changes in total magnetic intensity from changes in the other magnetic components [30,68–70,73]. Thus, it remains to be clearly established whether this component of the Earth’s magnetic field is actually detected and used by birds in a navigational context.

Our current discovery that magnetic inclination and magnetic declination alone can be used by birds to determine their approximate position is in line with a preceding study showing that experienced reed warblers can use magnetic declination as part of their map [31]. Interestingly, magnetic inclination is used by birds to distinguish between ‘polewards’ and ‘equatorwards” directions [13] as part of their magnetic compass sense. In birds the magnetic compass sense is proposed to be based on a radical pair sensory mechanism [74], which involves reversible, light-dependent chemical reactions inside the retina of the birds’ eyes, with the yield of these reactions depending on the alignment of a specific type of molecule (cryptochromes) relative to the Earth’s magnetic field axis [75–82].

While birds respond to magnetic inclination and magnetic declination in a compass context, current evidence suggests that a magnetic sense innervated via the trigeminal nerve is required to provide positional magnetic information [34,35]. The results of pulse remagnetizations are commonly interpreted as evidence that it is based on magnetic particles [83,84]. The structure and location of this sensory system are currently unclear [85], although the link to the trigeminal nerve suggests that the beak is a likely location in birds [86–88]. It is, therefore, possible that even though magnetic inclination and magnetic declination are associated with the magnetic compass sense, in the case of map navigation, one or both of these cues are detected by a separate trigeminal-based ‘magnetic map sense’. Given that the apparent sensitivity of the radical pair-based ‘magnetic compass sense’ in birds to inclination is about 3–5° at best [89,90], it is possible that this is not precise enough to resolve spatial locations for navigation. It should also be borne in mind though, that magnetic inclination and magnetic declination are human constructs to describe certain components of the magnetic field based on the way we measure and describe them. If the birds are using sensory systems with different characteristics to detect directions and locations, they may not perceive magnetic inclination and magnetic declination as the same components in these different contexts. On the other hand, it remains to be determined if a magnetic particle-based sense is used by birds to perceive magnetic inclination at all. In principle, if it has properties comparable to a three-axis magnetometer, then this would be possible through comparing changes in horizontal and vertical intensity. It thus remains important to understand the properties of the proposed magnetic particle-based sense to understand what aspects of the magnetic field it may perceive and whether these can be used for navigation.

In newts, it has been suggested that the radical pair-based ‘magnetic compass sense’ provides a spatial reference for a magnetic particle-based ‘magnetic map sense’, which would be required to detect the magnitude of the cues [91,92]. This is based on different effects of wavelength of light on the orientation of newts exhibiting ‘shoreward’ orientation versus homing [92]. In birds however, there is currently no clear evidence of such a difference occurring between the response to different wavelengths of light of juveniles exhibiting compass orientation [93] and adults that are, in principle, exibiting map navigation [81]. In order to fully clarify how the two putative magnetic senses may or may not interact in a navigational context, further targeted research studies will be needed.

The apparent use of magnetic declination as a positional cue also raises the question of how it is determined. It is known that birds have a time-independent star compass based on locating the centre of celestial rotation [11,94–96], which could provide the geographical reference throughout the migratory flight under clear conditions. Interestingly, migratory birds have been shown to be less likely to take off under cloudy conditions [97] and seem less likely to show a compensatory re-orientation for displacements [98]. Alternatively, it has been shown in a number of experiments that a magnetic compass may be calibrated by sunset cues [99], maybe related to polarized light cues [100], which could also provide a geographical reference for declination. However, attempts to show this phenomenon in other species, particularly of the Palaeartic-African migration system, have not yet been successful [101–103], which raises questions about its validity for the reed warbler. Nevertheless, further experiments should establish how magnetic declination is determined by birds. The use of magnetic declination also has potential implications for other experiments using magnetic coils. If coils are poorly calibrated, inadvertent small shifts in magnetic declination could occur which could have very significant effects on the direction chosen by the birds in Emlen funnels and thus may confound experimental results.

## 5. Conclusions

Based on our current results, it appears that birds can use a combination of magnetic inclination and magnetic declination to locate their position, even when the values of these cues do not match the geospatial variation of the total intensity of the Earth’s magnetic field. This suggests that a majority rule may apply, or possibly that the total intensity of the magnetic field does not form a part of the navigational map of birds. Birds use magnetic inclination and magnetic declination to determine direction during migration. Thus, it seems that cues that are associated with birds’ magnetic compass are also incorporated into their navigational (magnetic) map. Given that naive migratory songbirds use inherited directions as part of their clock-and-compass programme to reach their winter grounds during their first migration [14,56,104], it is conceivable that they learn the relative behaviour of their magnetic compass and their celestial compasses (e.g. their sun and/or star compass) in their geospatial context as they move along their migration route in order to establish such a navigational (magnetic) map. Whether the total intensity represents a redundant magnetic map cue and how birds perceive the different magnetic field components in different contexts still remains to be established.

## Data Availability

Data and an R script are available via Figshare: Data: [44]. R script: [53]. Supplementary material is available online [105].
